# A deep learning wearable-based solution for continuous at-home monitoring of upper limb goal-directed movements

**DOI:** 10.3389/fneur.2023.1295132

**Published:** 2024-01-05

**Authors:** Adonay S. Nunes, İlkay Yildiz Potter, Ram Kinker Mishra, Paolo Bonato, Ashkan Vaziri

**Affiliations:** ^1^BioSensics LLC, Newton, MA, United States; ^2^Department of Physical Medicine and Rehabilitation, Harvard Medical School Spaulding Rehabilitation Hospital, Boston, MA, United States

**Keywords:** deep learning, stroke diagnosis, upper limb function, accelerometer, wearables

## Abstract

**Introduction:**

Monitoring upper limb function is crucial for tracking progress, assessing treatment effectiveness, and identifying potential problems or complications. Hand goal-directed movements (GDMs) are a crucial aspect of daily life, reflecting planned motor commands with hand trajectories towards specific target locations. Previous studies have shown that GDM tasks can detect early changes in upper limb function in neurodegenerative diseases and can be used to track disease progression over time.

**Methods:**

In this study, we used accelerometer data from stroke survivor participants and controls doing activities of daily living to develop an automated deep learning approach to detect GDMs. The model performance for detecting GDM or non-GDM from windowed data achieved an AUC of 0.9, accuracy 0.83, sensitivity 0.81, specificity 0.84 and F1 0.82.

**Results:**

We further validated the utility of detecting GDM by extracting features from GDM periods and using these features to classify whether the measurements are collected from a stroke survivor or a control participant, and to predict the Fugl-Meyer assessment score from stroke survivors.

**Discussion:**

This study presents a promising and reliable tool for monitoring upper limb function in a real-world setting, and assessing biomarkers related to upper limb health in neurological, neuromuscular and muscles disorders.

## Introduction

Objective and quantitative assessment of lower and upper limb movement and functions can facilitate early detection, disease progression monitoring, and development of personalized treatment plans for individuals with neurological disorders. Goal-directed movements (GDMs) are the atomic components of upper limb movements, and the movement patterns depend on the planned motor commands with hand trajectories toward specific target locations (1). In GDMs, the central nervous system (CNS) coordinates between multiple muscle groups to work together in a specific sequence and timing to achieve a desired outcome. (2). The CNS receives sensory information about the goal and the environment and uses this information to plan and execute the movement. This process involves several stages, including sensory processing, motor planning, motor programming, and execution. GDMs are a crucial aspect of daily life for carrying out tasks such as reaching, grasping, and manipulating objects. For example, stroke can adversely affect GDMs in multiple ways, including motor impairments, sensory processing deficits, and cognitive deficits. In stroke rehabilitation, remote and quantitative assessment of GDMs is essential for the clinicians to assess the patient’s progress towards achieving functional goals (3–5).

Accelerometers provide a convenient way to measure upper limb movements and can be used to measure different movement patterns by detecting changes in acceleration. Previous methods rely on activity counts to measure upper limb function, usually by counting the number of zero crossings in the acceleration signal (6–8). However, activity counts provide an overall measure of upper limb movements, but are unable to differentiate between purposeful and non-purposeful movements, and do not provide information about the quality or complexity of the movements being performed (9–11). In contrast, GDM specifically measures upper limb function. Previous research has shown that GDM tasks can detect early changes in upper limb function in neurodegenerative diseases and can be used to track disease progression over time (12–15). Accelerometer data can provide a cost-effective solution to measure GDMs for measuring upper limb function and health in neurodegenerative, neuromuscular and muscle diseases.

Automated assessments of GDM have advantages over manual assessments, including reducing the potential for human error and subjectivity, offering more frequent and convenient assessments, tracking disease progression, detecting subtle changes in movement, and being more cost-effective. These benefits are particularly important for neurorehabilitation settings where precise and reliable neuromotor assessments are critical for patient outcomes, and for conditions such as Parkinson’s disease and ALS where frequent and accurate assessments can help with identifying changes in function and inform treatment decisions. Recent works thus explored machine learning approaches to automatically detect GDM from sensor measurements towards stroke rehabilitation applications (16–20), with ensemble models such as XGBoost attaining the best prediction performance (17). While deep learning models in this setting have also been tested (21–23), these works have been limited to traditional convolutional neural network architectures. In broader classification applications on multivariate time-series data such as multi-channel accelerometer measurements, state-of-the-art deep learning models comprise transformer architectures (24) and improved convolutional architectures with model-specific explainability (25).

Motivated by these studies, we developed an automated method of detecting GDM from wrist-worn accelerometer data via three approaches comprising both shallow and deep models using the data previously reported in (16). In classifying windowed accelerometer data as GDM or non-GDM, we demonstrated that a state-of-the-art deep learning model outperforms existing shallow models designed for stroke rehabilitation applications. We further validated the utility of detecting GDM by extracting features from GDM periods and using these features to predict whether the measurements are collected from a stroke survivor or a negative control participant. In addition, the features were also used to predict the Fugl-Meyer Assessment [FMA, (26)] score, a stroke-specific performance-based impairment index, from stroke survivors. The prediction performance was compared to the performance from models that used the entire recordings to extract the features, rather than only GDM periods.

## Methods

### Dataset

The accelerometer and label data in this study was previously used in (16). The reader can refer to the previous publication for greater detail. Here a summary is presented.

30 participants were recruited for the study, from which 20 were stroke survivors (mean 54.4, SD 10.1 years old; time since stroke mean 4.6, SD 5.5 years; FMA average 37, SD 8), and 10 controls (age 53.8, SD 11.4 years old). Stroke survivors were recruited from Spaulding Rehabilitation Hospital (SRH) inpatient and outpatient units. All subjects provided written informed consent and the study was approved by the SRH Institutional Review Board.

### Study design

Participants wore a six-axis inertial measurement unit (IMUs, Shimmer Research, Ireland) on each wrist and performed tasks resembling different types of ADL. Specifically, participants performed unimanual, bimanual and passive tasks. Table 1 explains the performed tasks for each category. Each motor task was repeated 3 times and therapist scripted and timed all the tasks. The entire experiment was videotaped and synchronized with the sensor data.

**Table 1:** Description of the tasks for each type of movement performed while accelerometer data was recorded.

Type	Task
Unimanual (affected limb)	Drink from a can
	Turn a key in a lock
	Hair brushing
Bimanual	Pick up pen from desk, remove the cap, and place it back
	Pick up a box and bring it to the knees
	Fold a hand towel
Passive	Walk
	Stand up without using arm for bracing
	Ascend and descend stairs
Task free	Periods without goal directed movements while accelerometer data is recorded

### Data labeling

Sensor data was labeled by inspecting the videos in segments of 2.5 s. Each segment was labeled as unimanual, bimanual, passive or task-free. During unimanual movements, the opposite side sensor data was labeled as task-free. For the GDM detection task, bimanual and active-side unimanual were categorized as GDM movements and the passive-side unimanual, passive and task-free were categorized as non-GDM.

### Data analysis

The six-axis IMU has an accelerometer that detects acceleration and a gyroscope that measures rotation. For this study, only the accelerometer was used, given that the energy consumption of the accelerometer is lower than gyroscope (16). That makes it better suited for long-term remote monitoring.

Triaxial acceleration time series for each wrist IMU were band-passed between 0.1 and 12 Hz with a 4th order butterworth filter to remove the inertial gravity component and high frequency activity, and then down sampled to 25 Hz. Triaxial velocity data was estimated by integrating acceleration data. Then, the same band-pass filter was applied. High frequency cut-off did not discard any activity measurements, as typical movements attain at most 10 Hz frequency (27).

A sliding window of 3 s with 70% overlap was used to segment the data for training the classification model, as these parameters ensure enough data to capture a GDM, and with the overlap more data is obtained for the model to better generalize. At each window, if one third of the time points were labeled as GDM, the entire window was labeled as GDM movement. For each IMU, data were windowed separately. In total, 49,254 6-dimensional time windows were extracted, with 21% labeled as GDM. Optimal window size and overlap were determined from validation set performance, as explained below.

A leave-one-subject-out (LOSO) cross-validation was used to test model performance. Ten percent of the training subjects for each cross-validation split were further held-out as validation data and remaining training subjects were used for training. Data normalization was performed by subtracting the population mean from each sample and dividing the resulting values by the population standard deviation, where mean and standard deviations were estimated from the training set for each split. As the distribution of GDM vs. non-GDM windows was imbalanced, the training objective for each class was weighted with the ratio of the other class in training. The validation set was used for early-stopping of model training and for finding the optimal probability threshold to differentiate the two classes. The optimal threshold was found by taking the geometric mean of the true positive rate and the true negative rate (28).

We tested three state-of-the art classification methods for multivariate time-series signals to differentiate GDM activity windows from others. The models used were from the python package TSAI (29). To begin with, we employed a decision-tree classifier using gradient boosting termed XGBoost. XGBoost was designed particularly to tackle small and imbalanced datasets via ensembling and pruning (30). Moreover, it outperformed other non-deep learning models in multivariate time-series classification tasks, including measuring activities of daily living (17), and attained comparable performance to deep learning (24).

One of the deep learning classifiers we employed was a transformer model (24). To initialize the transformer classifier weights, an autoencoder model comprising a transformer encoder and a fully-connected decoder was trained over the multivariate time-series samples in the training set by minimizing a masked reconstruction error loss in an unsupervised manner. The transformer encoder architecture followed the well-known design by Vaswani et al. (31), with the modifications of fully-trainable positional encoding, batch normalization and the same hyperparameters as optimized by Zerveas et al. (24). Following unsupervised pre-training, the decoder was replaced by a fully-connected layer with a scalar output and sigmoid activation. The resulting transformer classifier was fine-tuned by minimizing a cross-entropy loss to classify each input window as GDM or non-GDM. As transformer training took significantly longer than other methods, we tested this model via 5-fold cross validation with stratified partitioning over stroke survivors and controls.

The other deep learning approach was an eXplainable Convolutional Neural Network, which aggregated features from 1D and 2D convolutions with model interpretability via Gradient-weighted Class Activation Mapping [XCM, (25)]. This model was designed for multivariate time-series classification and has been shown to outperform other models when classifying physiological signals. In our application, XCM also performed the best out of all competing methods, as we discuss below.

To demonstrate the utility of the trained model beyond GDM detection, we tested if features from the inferred GDM periods from both hands can better classify stroke survivors and controls compared to features extracted from all the recordings. In addition, we tested if FMA can be better predicted with GDM features from both hands compared to features from the entire recordings. Total of 28 features were estimated from the acceleration and velocity time series. The time series were either tri-axial or magnitudes over the three axes. Tri-axial measures included the correlation between axis pairs and the number, mean length and length entropy of zero crossing segments. Measures based on magnitude were the minimum, maximum, median, root mean square, the domain frequency over energy (the peak frequency divided by the total spectrum), skewness, kurtosis, and entropy. Elastic net regression was used to classify or predict, and LOSO validation was used to quantify the model performance.

## Results

In total, 1,830 activity periods were labeled, with 961 task-free, 79 passive, 249 unimanual and 541 bimanual activities. This corresponded to 608 h of task-free activity, 44.5 h of passive activity, 56 h of unilateral movements and 138.5 h of bilateral movements. The stroke participants represented 72.5% of the data. As only the affected side for stroke patients or the non-dominant side for controls was used during unimanual activities, the passive side was labeled as non-GDM for these activities. When a sliding window of 3 s with a 70% overlap was applied, the total number of windowed time series used for training and testing were 49,254, out of which 21% were labeled as goal-directed movements. This includes both right and left side IMU accelerometer data.

**Figure 1:**
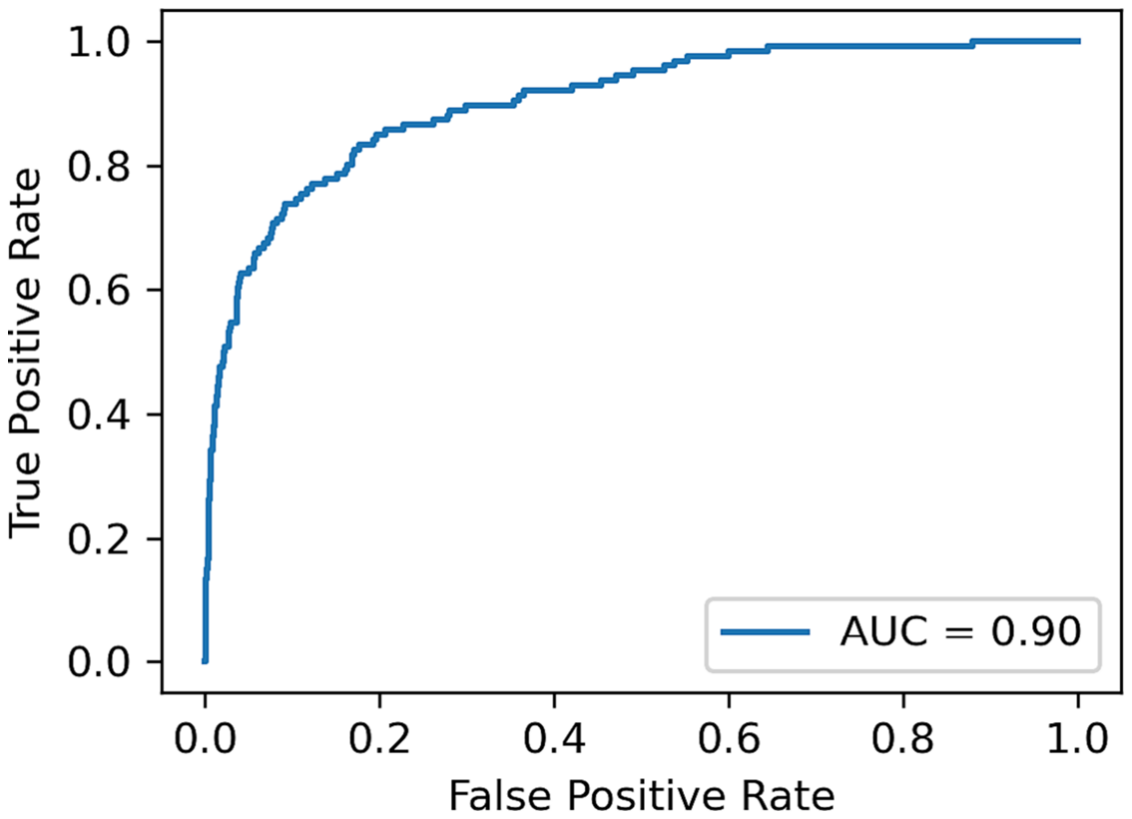
ROC-AUC for differentiating GDM from non-GDM movements.

A LOSO cross validation was used to assess GDM activity detection performances of XGBoost, Transformer and XCM models. The model performance was calculated over the test set of each cross validation split with respect to Area under the receiver operating characteristic (AUC), sensitivity, specificity and F1 score metrics. Average AUC, sensitivity, specificity and F1 score metrics for all methods are reported in Table 2. XCM outperformed other shallow and deep models and attained 0.90 AUC, 0.81 sensitivity, 0.84 specificity and 0.82 F1 score. The receiver operating characteristic curve (ROC) for the XCM model is visualized in Figure 1 for Positive class, i.e., GDM labels, includes the active hand during unimodal movements and both hands during bimanual activities, while non-GDM labels include the passive hand during unilateral movements and passive activities such as walking and standing up from a chair without armrest, and task-free periods where subjects are not performing either task. To further analyze the performance of the XCM model during different tasks, the accuracy in different tasks was calculated separately for the stroke survivors and controls, with results presented in Table 3. For both groups, accuracy calculated over different sides were close to each other, showing promise for generalization performance of XCM.

**Table 2:** GDM activity detection performance.

Method	AUC	Balanced accuracy	Sensitivity	Specificity	F1 score
XGBoost	0.83	0.76	0.75	0.77	0.75
Transformer	0.83	0.77	0.78	0.75	0.76
XCM	0.90	0.83	0.81	0.84	0.82

**Table 3:** Model performance for different tasks in stroke survivor and control groups.

Side	Bimanual	Unimanual	Passive	Task free
Stroke participants				
Affected	0.81	0.82	0.83	0.79
Unaffected	0.76	0.73	0.85	0.77
Control participants				
Dominant	0.94	0.89	0.99	0.81
Non-dominant	0.91	0.76	1.0	0.83

An elastic net logistic regression model (32) was trained to differentiate between stroke survivors and controls using features extracted solely from periods labeled as GDM by the XCM model and using features extracted from the entire recording. A LOSO cross-validation was used and the performance was assessed with average accuracy, sensitivity and specificity. The model using GDM features outperformed the setting of using entire recordings, with a balanced accuracy of 0.9, sensitivity of 1 and specificity of 0.8, compared to an accuracy of 0.75, sensitivity of 1 and specificity of 0.5. This indicates that features learned from GDM windows have more information to differentiate between groups, showing further promise for rehabilitation applications. Table 4 shows the group statistics for GDM features and their statistical significance.

**Table 4:** GDM extracted features group statistics.

	Stroke survivors			Control participants				
Feature	Mean	±	SD	Mean	±	SD	Cohen’s D	*p*-value
Acc min (m/s2)	0.21	±	0.1	0.5	±	0.11	−2.81	<0.001
Acc median (m/s2)	2.04	±	0.52	3.1	±	0.39	−2.2	<0.001
Acc RMS	2.87	±	0.58	4.14	±	0.59	−2.18	<0.001
Acc crossing entropy	1.04	±	0.02	1	±	0.02	2.28	<0.001
Vel skewness	0.86	±	0.24	0.37	±	0.11	2.34	<0.001
Vel crossing entropy	1.07	±	0.01	1.05	±	0.01	2.3	<0.001
Vel median (m/s)	31.79	±	7.49	46.76	±	5.82	−2.14	<0.001
Acc entropy	6.55	±	0.44	5.87	±	0.18	1.84	<0.001
Vel RMS	41.76	±	8.28	55.85	±	6.45	−1.82	<0.001
Vel entropy	6.59	±	0.46	5.89	±	0.17	1.81	<0.001
Vel kurtosis	0.53	±	0.77	−0.55	±	0.17	1.69	<0.001
Vel crossing number (*n*)	75.61	±	43.6	32.13	±	5.1	1.21	0.004
Vel min	4.21	±	1.54	5.92	±	1.13	−1.2	0.004
Acc crossing average length (samples)	8.82	±	2.1	11.2	±	2.12	−1.13	0.007
Acc crossing number (*n*)	494.36	±	376.97	159.91	±	26.59	1.08	0.010
Vel corr XZ	−0.02	±	0.15	−0.17	±	0.14	1.02	0.014
Vel max (m/s)	96.22	±	13.46	107.81	±	11.65	−0.9	0.028
Acc corr XZ	−0.03	±	0.09	−0.1	±	0.09	0.81	0.045
Acc corr YZ	−0.31	±	0.11	−0.22	±	0.12	−0.81	0.046
Acc max (m/s2)	10.78	±	2.09	12.47	±	2.17	−0.8	0.048
Acc kurtosis	5.81	±	3.88	3.29	±	1.6	0.76	0.060
Acc skewness	1.67	±	0.46	1.38	±	0.22	0.73	0.071
Vel corr YZ	−0.55	±	0.21	−0.48	±	0.19	−0.37	0.347
Acc dom freq over energy	0.00013	±	0.00015	9.37E-05	±	2.78E-05	0.32	0.421
Acc corr XY	0.0137	±	0.08	0.00078	±	0.08	0.16	0.690
Vel crossing average length (samples)	44.83	±	2.82	45.27	±	3.6	−0.14	0.718
Vel corr XY	0.03	±	0.13	0.01	±	0.1	0.14	0.723
Vel dom freq over energy	5.60E-07	±	1.87E-07	5.7E-07	±	1.42E-07	−0.07	0.860

Acc, acceleration; Vel, velocity; corr, correlation; dom, domain; freq, frequency.

**Figure 2:**
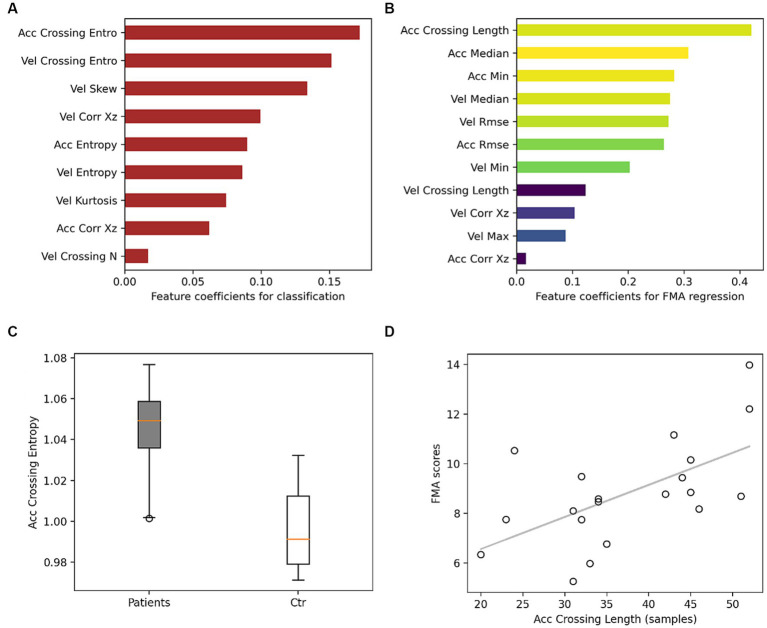
Models feature coefficients. **(A)** Feature coefficients for classifying stroke survivors from controls. Red indicates that the mean in the stroke group is higher than controls. **(B)** Feature coefficients for regression FMA, colors from yellow to dark blue indicate larger to smaller correlation between the feature and FMA scores. **(C)** Boxplot for the feature with the largest contribution in the classification. **(D)** Scatter plot between FMA scores and the largest feature contributing to FMA prediction with a correlation coefficient of 0.6. Acc, acceleration; Vel, velocity; entro, entropy; *N*, number.

Figure 2A shows the largest logistic regression coefficient magnitudes corresponding to features extracted from GDM periods that contributed most to classification. The regression performance for FMA scores was also higher when using GDM features, with a mean absolute error of 6.9 and an explained variance of 21%, compared to a mean absolute error of 8 and an explained variance of 0.6%. Figure 2B shows the largest elastic net linear regression coefficient magnitudes for the most important features in predicting FMA scores.

## Discussion

In this study, we trained three machine learning models on accelerometer data to detect goal-directed movements during tasks resembling activities of daily living. The best performing deep learning model achieved an AUC of 0.90 and a balanced accuracy of 0.83, which is a promising result for this type of task. Previously, (16) achieved an 0.87 AUC, a true positive rate of 0.79 and a true negative rate of 0.78, training on data from unimanual, bimanual and passive tasks using a Random Forest classifier. Our deep learning approach via XCM not only outperforms Random Forest in GDM detection with respect to three classification metrics (Table 2), but also does not discard task-free recordings. Thus, the proposed model has been trained on and can attain high accuracy over a wider range of ADL, and thus, is more generalizable to real-life applications.

An interesting aspect of our study is that 60% of the dataset comprised stroke survivors. The performance of the model was slightly lower for this group compared to the control group. This could be due to the nature of movements in stroke survivors, which can be more variable and less well-defined than in healthy individuals. An additional possible explanation for this discrepancy is that stroke survivors may exhibit idiosyncratic patterns of movement that are specific to individual types of stroke and locations of neurological damage. These individual differences may have impacted the model’s ability to accurately detect GDM. Therefore, future studies may need to account for these individual differences in order to improve the accuracy and generalizability of our approach. This could include collecting data from a larger and more diverse sample of stroke survivors, as well as analyzing the effects of different types of stroke and locations of neurological damage on movement patterns.

The results in this study have important implications in clinical care for individuals with neurological, neuromuscular and muscle disorders, as well as for the field of rehabilitation, as automatic detection of goal-directed movements can be used to monitor patients’ progress and provide feedback to clinicians. While previous studies have used various algorithms to detect upper limb function (33–36), our use of state-of-the-art deep learning models is a relatively new approach to specifically detect GDM. Compared to previous studies, our model achieved comparable or even better results. For example, (37) reported an accuracy of 88% in controls and 70% in stroke survivors for detecting three types of arm movements using accelerometer data, while (38) reported an accuracy of 84.5% for detecting hand gestures using electromyography and accelerometer data.

Our study goes beyond simply detecting GDM and demonstrates the usefulness of extracting features from detected GDM periods. Specifically, we used these features to predict whether the participant was a stroke survivor or a control participant and to predict the FMA score, which is a stroke-specific performance-based impairment index. The model trained on features from GDM periods outperformed the model trained on features from the entire recording. Furthermore, we found that the performance of regression of FMA scores was also higher when using GDM features. These results suggest that not only can our deep learning model accurately detect goal-directed movements, but also the features extracted from these movements have additional information that can be used to differentiate between stroke survivors and control participants and to predict stroke-specific impairment. The most important features for classification and regression were related to zero crossings and indicate movement discontinuities with acceleration or velocity changing directions.

While wrist-worn accelerometers may not capture all movement nuances such as rotation and elevation, they offer a cost-effective means for continuous monitoring. This approach, although not perfect, holds promise for applications like remote patient monitoring and rehabilitation. By continuously recording accelerometer data and then sending them to the cloud for processing, meaningful features from GDM can be extracted. These features can be valuable for clinical practitioners and as outcomes in clinical trials.

In conclusion, our study shows that a deep learning model can achieve high levels of accuracy for automatic detection of goal-directed movements, even with data collected from stroke survivors, and suggests that deep learning models are a good candidate for monitoring upper limb function using wrist-worn accelerometers. Our results have important implications for the field of remote patient monitoring and rehabilitation.

## Data availability statement

The datasets generated and analysed during the current study are proprietary and not publicly available due to being supported by an NIH Small Business grant award. As outlined in the 2023 NIH Data Management & Sharing Policy, “SBIR and Small Business Technology Transfer (STTR) recipients may retain the rights to data generated during the performance of an SBIR or STTR award for up to 20 years after the award date, per the SBIR and STTR Program Policy Directive but are available on reasonable request from the corresponding author.

## Ethics statement

The studies involving humans were approved by the SRH Institutional Review Board. The studies were conducted in accordance with the local legislation and institutional requirements. The participants provided their written informed consent to participate in this study.

## Author contributions

İY: Conceptualization, Formal analysis, Methodology, Writing – original draft. RM: Formal analysis, Writing – original draft. PB: Project administration, Resources, Writing – review & editing. AV: Conceptualization, Methodology, Supervision, Writing – original draft. AN: Conceptualization, Formal analysis, Methodology, Writing – original draft.
